# Active Commuting among K-12 Educators: A Study Examining Walking and Biking to Work

**DOI:** 10.1155/2013/162731

**Published:** 2013-09-08

**Authors:** Melissa Bopp, Tanis J. Hastmann, Alyssa N. Norton

**Affiliations:** ^1^Department of Kinesiology, The Pennsylvania State University, University Park, PA 16802, USA; ^2^Department of Physical Education, Exercise Science and Wellness, the University of North Dakota, Grand Forks, ND 58202, USA

## Abstract

*Background*. Walking and biking to work, active commuting (AC) is associated with many health benefits, though rates of AC remain low in the US. K-12 educators represent a significant portion of the workforce, and employee health and associated costs may have significant economic impact. Therefore, the purpose of this study was to examine the current rates of AC and factors associated with AC among K-12 educators. *Methods*. A volunteer sample of K-12 educators (*n* = 437) was recruited to participate in an online survey. Participants responded about AC patterns and social ecological influences on AC (individual, interpersonal, institutional, community, and environmental factors). *t*-tests and ANOVAs examined trends in AC, and Pearson correlations examined the relationship between AC and dependent variables. Multiple regression analysis determined the relative influence of individual, interpersonal, institutional, community, and environmental levels on AC. *Results*. Participants actively commuted 0.51 ± 1.93 times/week. There were several individual, interpersonal, institutional, community, and environmental factors significantly related to AC. The full model explained 60.8% of the variance in AC behavior. *Conclusions*. This study provides insight on the factors that determine K-12 educators mode of commute and provide some insight for employee wellness among this population.

## 1. Background

 Regular physical activity can improve the health and overall well-being of Americans of all ages [1]. However, current estimates indicate that the majority of adults aged 18–64 do not meet the recommended physical activity requirements of 150 minutes each week [2]. Physical activity provides numerous health benefits and reduces the risk for chronic disease morbidity and mortality. Additionally, there are a number of noted economic implications for low rates of physical inactivity [1, 3–6].

The benefits of overall physical activity are well documented. In addition to the obvious health benefits, engaging in physical activity is associated with less employee turnover [7], happier employees [8], reduced absenteeism [9], and reduced job stress [10]. Most importantly, physical activity is associated with reduced illness-related absenteeism [11]. Similarly, active transportation to and from school or a workplace, known as active commuting (AC), also provides numerous health benefits [12], including a reduced risk of obesity [13], cardiovascular disease [14], and all-cause mortality [15]. Despite these well-documented benefits, rates of AC remain low in the United States. Data from the National Health Interview Survey have shown that only 28% of individuals report walking anywhere as a mode of transportation [16], while Hu and Reuscher [17] have found that only 2.8% of individuals report walking to work. These rates remain quite low compared with Europe and Australia [18]. Comprehensively understanding the factors that influence AC will allow for targeting and tailored interventions to increase participation in this behavior.

 Social ecological frameworks [19, 20] offer a comprehensive approach to understanding and changing physical activity behavior. Several studies have highlighted individual level influences on AC, including beliefs and attitudes [21–23], habit strength [24], and demographics [25]. Other research has examined social support [26] or the social environment [23] as possible influences on AC. Kaczynski and colleagues [26] noted that the presence of physical support, measured by the availability of bike parking, storage policies, showers, or lockers at the workplace was associated with a threefold increase in actively commuting to work. Similar to other studies examining how the built and natural environment can influence physical activity participation [19, 27–29], environmental influences on walking and biking to work are also well documented [22, 23, 25, 30, 31]. 

 Across the United States, there are more than 130,000 K-12 schools, serving more than 55 million children [32]. To serve these children, there are more than 3.2 million teachers in the US, with many hundreds of thousands of more support staff and administrators. In 2007-2008, it was estimated that of the $506.8 billion spent in public schools in the US, $303.2 billion were related to instruction and personnel, making employees a valuable resource in schools across the country. State and local governments shoulder the bulk of the financial burden with public education [32], and current economic conditions have resulted in some significant challenges in meeting the needs of the community with declining resources. One way to potentially reduce the enormous costs associated with educators and personnel would be to improve their health. As previously discussed, employees who are active have greater physical and mental health and less illness-related absenteeism compared to their lesser active counterparts [9, 11]. This information is corroborated by van Amelsvoort and colleagues, who showed that workers who engaged in leisure time physical activity twice or more per week reported significantly less sick absence compared to inactive workers [33]. One way to reduce illness-related absenteeism may be to increase employee active commuting. Therein the health and well-being of educators present some significant community level concern. 

 Although there has been evidence that AC has positive health effects on adults, there has not been much research on K-12 educators actively commuting to their workplace. Therefore, the purpose of this study was twofold: first, to analyze the patterns of AC and factors associated with teachers' AC and second, to extrapolate how these factors could improve the likelihood of teachers walking or riding their bike to work. 

## 2. Methods

This cross-sectional survey was delivered online using the Qualtrics software program (Provo, UT, USA) from June–December 2011. This study was approved by the Institutional Review Board at Pennsylvania State University. 

### 2.1. Participants

Individuals over the age of 18 years, employed full- or part-time outside of the home and physically able to walk or bike were eligible to take part in the survey. Recruitment took place primarily in the mid-Atlantic region of the US (PA, OH, WV, MD, NJ, and DE). Further details of the survey and non-K-12 educators are available elsewhere [34]. Websites of K-12 school districts in medium-large cities were examined for employee email addresses, and individuals were contacted directly via email. The email invitation description invited people to take part in a “commuting survey” in order to limit a volunteer bias of people who may have been more interested in active commuting methods. Survey invitations were emailed to *n* = 2416 K-12 school district employees. Among the invitees, *n* = 437 completed the survey, for a response rate of 18.1%.

### 2.2. Instruments

#### 2.2.1. Individual Level

*Commuting Patterns*. Participants reported the number of times per week that they walked, biked, drove, and took public transportation to and from work. The number of individual trips via walking and biking was summed for number of active commuting trips/week. 

*Demographics and Medical*. Participants reported their age, race/ethnic group (dichotomized as non-Hispanic White/other), marital status (dichotomized as married, partnered/not married, or partnered), number of children, sex, and number of cars in the household. Participants indicated from a list how many chronic diseases they had and reported their height and weight for body mass index (BMI) calculations. Respondents were asked to rate their current health status with a 5-point Likert scale from 1 (poor) to 5 (excellent). 

*Self-Efficacy*. Participant's confidence with their cycling skills in urban areas was assessed with a single item using a 4-point Likert scale (1 = not at all confident to 4 = very confident). 

*AC Behavioral Beliefs*. Respondents indicated their agreement using a 7-point Likert scale (1 = completely disagree to 7 = completely agree) with 13 statements about AC. Eight were related to AC and their physical or mental health (e.g., AC helps me control my weight can help me to relieve stress), and five were related to other AC benefits (e.g., AC is good for the environment helps me to save money). A total score was computed for all 13 items. This measure was based on a previously tested scale [35] and demonstrated excellent reliability in the present study (*α* = 0.91). 

*Perceived Behavioral Control for AC*. Participants indicated their agreement using a 7-point Likert scale (1 = completely disagree to 7 = completely agree) with six statements about why AC is difficult (e.g., AC is difficult because I am not committed to it because I am too tired) [36]. A total score was computed for the six items, and the scale showed good reliability (*α* = 0.84). 

#### 2.2.2. Interpersonal

*Coworker and Spouse AC Participation and Normative Beliefs*. Participants responded with a Likert scale (1 = strongly disagree to 5 = strongly agree) to a question about their coworkers' AC behavior: “Most of my coworkers walk or bike to/from work.” Respondents also reported the number of times/week their spouse walked or biked to/from work. Coworker and spouse normative beliefs were assessed separately using a 5-point Likert scale to measure an individuals' level of agreement with four statements about their spouse/coworkers influence on their mode of travel to work. Items asked separately for spouse and coworkers but were worded identically. Items included “My spouse and I discuss issues related to walking and biking to work,” “I value what my spouse thinks about the way I travel to/from work,” “I have an opinion on the way my spouse travels to/from work,” and “My spouse influences my choice on how I travel to/from work.” Items were summed separately for spouse and coworkers and had good reliability (coworkers *α* = 0.76, spouse *α* = 0.83). 

#### 2.2.3. Institutional

*Worksite Related.* Participants were asked to report their employer's size and number of employer supports for AC (yes/no) from a list of seven items (incentives for AC, events related to AC, and flexible work hours, bike storage policies, bicycle parking, locker rooms, flexible dress code), which were summed. Participants indicated how much they perceived their employer supported AC using a 5-point Likert scale (1 = strongly disagree to 5 = strongly agree). Perceived problems for parking at work were assessed with three items about a lack of availability, high cost, and difficulty of parking. Parking items were summed with a greater score indicating more problems associated with parking.

#### 2.2.4. Community

*Community Factors*. Participants reported (yes/no) on the availability of three supports for bicyclists in their community (e.g., bike racks on buses, covered bike parking, “share the road” signs), which were summed. Participants to indicate their agreement with five statements about perceived support for walking and biking using a 5-point Likert scale (1 = strongly disagree to 5 = strongly agree). Items were summed to create a perceived environmental supports score and included: town/city support for pedestrian or bicyclists issues, seeing others in their community walking/biking, and maintenance of sidewalks or bike lanes. Individuals were asked to rate their community's perceived pedestrian and bike friendliness from 1 (not pedestrian/bicycle friendly at all) to 5 (very pedestrian/bicycle friendly). Lastly, participants also indicated how long it would take them to walk or bike to work, dichotomized as ≤20 minutes and greater than 20 minutes. 

#### 2.2.5. Environmental

*Barriers*. Perceived environmental barriers were examined, with respondents rating the extent to which seven environmental features kept them from walking or biking to work (1 = strongly disagree to 5 = strongly agree). The items included a lack of on-street bike lanes, lack of off-street walking/biking paths, lack of sidewalks, speed/volume of traffic along route, perceived crime along route, difficult terrain, and bad weather.

### 2.3. Data Analysis

Basic descriptive statistics and frequencies were used to describe the sample. *t*-tests compared differences between older and younger participants. For categorical correlates of AC (e.g., gender), *t*-tests and analyses of variances (ANOVAs) were used to examine differences in rates of AC. For continuous variables (e.g., age, BMI, and perceived behavioral control), Pearson correlations were used to examine associations with AC. The variables with significant associations were included in a forced block entry multiple regression analysis to determine the relative influence of social ecological factors for AC. Specifically, five blocks of independent variables were forced into the model: individual, interpersonal, institutional, community, and environmental-level variables. All analyses were performed using IBM SPSS 20.0 (Armonk, NY, USA), and significance levels were set at *P* < 0.05.

## 3. Results

The characteristics of the sample are found in Table 1. Respondents were primarily middle aged (mean age 44.70 ± 11.11 years), female (79.9%), non-Hispanic White (93.4%), and overweight (BMI 26.40 ± 5.9 kg/m^2^). On average participants actively commuted 0.51 ± 1.93 times/week, drove 8.77 ± 3.19 times/week, and took public transit 0.04 ± 0.68 times/week. Only 8.7% of participants reported actively commuting one or more times/week. Pearson correlations for AC are shown in Table 2. 

### 3.1. Influences on Active Commuting

At the individual level, there were no differences for AC rates by sex, marital status, or race. BMI (*r* = −0.11, *P* = 0.04), number of cars in the household (*r* = − 0.18, *P* < 0.001), and perceived behavioral control (*r* = −0.44, *P* < 0.001) were negatively related to AC, while self-efficacy for biking skills (*r* = 0.14, *P* = 0.02) and AC beliefs (*r* = 0.13, *P* = 0.02) was positively related to AC. At the interpersonal level, spouse AC (*r* = 0.29, *P* < 0.001), spouse normative beliefs (*r* = 0.26, *P* < 0.001), and coworker normative beliefs (*r* = 0.24, *P* < 0.001) were positively related to AC. Amount of employer support (*r* = 0.14, *P* < 0.001) and perceived (*r* = 0.15, *P* < 0.001) employer support was positively related to AC at the institutional level. At the community level those reporting less than a 20-minute walk (*t* = 8.07, *P* < 0.001) or bike (*t* = 7.17, *P* < 0.001) time to work were more likely to AC than those reporting a longer travel time. A lack of on-street bike lanes (*r* = −0.15, *P* = 0.01), lack of off-street walking/biking paths (*r* = −0.20, *P* < 0.001), lack of sidewalks (*r* = −0.22, *P* < 0.001), speed and volume of traffic along route (*r* = −0.14, *P* = 0.01), difficult terrain (*r* = −0.18, *P* < 0.001), and bad weather (*r* = 0.13, *P* = 0.03) were all negative environmental factors associated with AC. Since perceived walk time and bike time were highly related, only walk time was used in the full model. 

The full model explained 60.8% of the variance in AC behavior (refer to Table 3). The individual block explained 42.0% of the variance (*F*(6,98) = 11.80, *P* < 0.001) with perceived behavioral control (*β* = −0.48, *P* < 0.001) as a negative predictor. The interpersonal level block (2nd block) explained an additional 11.2% of the variance (*F*(9, 95) = 11.99, *P* < 0.001), with spouse AC (*β* = 0.10, *P* = 0.001) as a positive predictor. The third block (institutional level) explained an additional 0.1% of the variance in AC (*F*(11, 93) = 9.65, *P* < 0.001) with no significant predictors. The fourth block (community level) explained an additional 2.9% of the AC variance (*F*(12, 92) = 9.83, *P* < 0.001) with walk time as a negative predictor (*β* = −0.18, *P* = 0.01). The final block (environmental level) contributed another 4.6% (*F*(18, 86) = 7.41, *P* < 0.001), with lack of sidewalks (*β* = −0.37, *P* = 0.03) and speed and volume of traffic along route (*β* = −0.30, *P* = 0.02) as negative predictors.

## 4. Discussion

 This study provides insight on the factors that determine whether K-12 educators choose to actively commute to work. The overall results of this study are important findings for the general health and well-being of educators and the individuals (i.e., students and staff) they influence. Few studies have examined AC rates among this occupational class, yet there are significant health and economic outcomes associated with these findings. These results provide a number of possible implications for school health and employee health in school districts. 

Public school districts providing health insurance are often saddled with the costs of rising healthcare expenditure, frequently accounting for a significant portion of the district's budget. For example, the State of New York reports that in 2007-2008 8.5% of an average school districts' expenditures were associated with health insurance [37]. Garrett and colleagues [6] have examined the direct costs to a health plan associated with physical inactivity. The findings suggested that 12% of mental health costs and 31% of chronic disease costs (colon cancer, cardiovascular disease, and osteoporosis) were attributable to a lack of physical activity participation. Actively commuting to work can increase the likelihood of individuals meeting current recommendations for physical activity [12, 27] which are associated with a decreased risk of chronic disease morbidity and mortality [1]. Olabarria and colleagues [38] showed significant positive health and economic outcomes when motorized trips were replaced by walking. Several reviews [39–41] have noted the cost-effectiveness of investments in worksite physical activity interventions, and school districts concerned with rising healthcare costs may consider interventions targeting AC as a method of improving health outcomes. 

In addition to the potential health benefits garnered from regular physical activity, teachers who actively commute to school could potentially influence their students to live an active and healthy lifestyle. Few studies have examined teachers influence on their student's physical activity behaviors, however based on Social Cognitive Theory, this link between teachers AC and student physical activity would be well supported. Specifically, teachers serve as a role model to student's and have the potential to increase their self-efficacy for physical activity [42]. Providing vicarious experiences, such as AC modeling, to increase motivation is a strategy teachers could use to promote PA among children, which is a growing health concern with high rates of childhood obesity and sedentary behavior [43]. 

 These findings in the present study indicated that the most influential factors for AC were individual level factors. Perceived behavioral control, AC beliefs, and self-efficacy for AC were significant predictors. It was apparent that the more barriers an individual endures, including lack of knowledge or skills or attitudes toward AC, the less likely they will actively commute. To address this barrier, there should be a focus on the individual level with theoretically based behavior-change strategies. Approaches could include goal setting, monitoring behavior, improving knowledge, and skills through educational approaches (e.g., instructional on health and financial reasons to bike to work, route selection, dealing with cargo and clothing, lighting, and traffic issues), all of which target self-efficacy and perceived behavioral control. Other individualized approaches could include teacher-tailored websites that offer encouragement, advice, and behavioral cues reminding teachers of the importance of their role modeling behavior. 

On an interpersonal level, there was a positive correlation between the number of times a week a teacher actively commutes to work and the number of times their spouse actively commutes. These results corroborated with a review by Panter and Jones [23] that examined associations between social support from family and friends and active travel which demonstrated that social support was positively associated with active travel and biking to work. Although not found in the current study, other studies have found an association between an individual's AC participation and their coworkers' AC [26]. This suggests that it is essential to address social support and social norms when targeting AC participation. This could include the involvement of family in education and skill building strategies or building social support within the worksite for AC behavior. 

 Sallis and colleagues [27] have emphasized that changing built environments and developing pedestrian- and bicyclist-friendly policies can have a long-term impact on the people in those places and are related to rates of chronic disease, hypertension, and obesity. They also reveal that physical activity levels are driven by different built environment features and policies, which was evidence in the current study, with distance, infrastructure, and safety as significant concerns related to AC. This reinforces the approach of targeting the environment to be more pedestrian and bike friendly to have an impact on the amount of daily physical activity levels people in the community would obtain. 

 Although there were significant findings related to teachers active commuting to work through this study, there were some limitations within the process. First of all, the surveys were conducted through self-report and subjective measures. Self-report questionnaires are not always accurate, whereas some individuals may not report truthful or precise answers. Secondly, the sampling strategy of the study was not random. Additionally, the individuals who volunteered to participate may have had a bias towards the institution or the topic of the study. There was also not a strong response rate; though due to the use of electronic email as our recruitment method, we were not able to ascertain if individuals received the invitation to participate in their “inbox” compared with their “junk mail” box. However, the study still provides insight on teachers actively commuting to work and provides a baseline for future studies.

AC to work is an important source of daily physical activity; however, only a small percentage of teachers in our sample walk or rode their bike to work each day. Active commuting can improve the overall health and well-being of teachers, in addition to significant economic outcomes for school districts, making it an important public health and community issue. Furthermore, the physical activity behaviors of teachers can influence the behaviors of their students and increase the amount of students active commuting to school targeting childhood inactivity and obesity issues.

## Figures and Tables

**Table 1:** Characteristics of the sample of K-12 educators (*n* = 437).

Variable	*n* (%)	Mean (SD)
Individual level		
Age		44.7 (11.1)
Sex		
Male	77 (20.1)	
Female	283 (79.9)	
Marital status (% married/partnered)		
Married/partnered	297 (79.8)	
Single, divorced, widowed	75 (20.2)	
Race/ethnicity		
Non-Hispanic White	327 (93.4)	
All other racial/ethnic groups	23 (6.7)	
Number of children		0.63 (0.96)
Body mass index (kg/m^2^)		26.4 (5.9)
Number of reported chronic diseases		0.62 (1.01)
Perceived health status (range: 1–5)		3.69 (0.80)
Number of cars in the household		3.26 (0.87)
Self-efficacy for bicycling skills (range: 1–4)		2.98 (1.53)
AC behavioral beliefs score (range: 13–91)		68.7 (13.66)
Perceived behavioral control for AC (range: 7–42)		29.06 (7.4)
Interpersonal level		
Spouse AC (times/week)		0.23 (1.4)
Spouse normative beliefs for AC (range: 4–20)		10.04 (4.07)
Perceived coworker AC (range: 1–5)		1.31 (0.61)
Coworker normative beliefs for AC (range: 4–20)		7.64 (3)
Institutional level		
Number of employer supports for AC (range: 0–7)		1.43 (1.42)
Perceived employer support for AC (range: 1–5)		2.03 (1.17)
Community level		
Perceived community support for AC (range: 5–25)		16.16 (4.82)
Perceived pedestrian friendliness for AC (range: 1–5)		3.27 (1.27)
Perceive bicycle friendliness for AC (range: 1–5)		3.07 (1.29)
Environment level (range 1–5)		
Lack of on-street bike lanes		3.15 (1.63)
Lack of off-street walking/biking paths		3.16 (1.62)
Lack of sidewalks		3.1 (1.64)
Speed and volume of traffic along route		3.36 (1.6)
Perceived crime along route		2.11 (1.43)
Difficult terrain		3.05 (1.54)
Bad weather		3.54 (1.45)

*Note*. AC: active commuting.

**Table 2:** Pearson correlations for active commuting and social ecological model variables.

Variable	*r*	*P*
Individual level		
Age	0.01	0.99
Body mass index (kg/m^2^)	−0.109	**0.04**
Number of reported chronic diseases	−0.017	0.72
Perceived health status (range 1–5)	0.14	**0.008**
Number of cars in the household	−0.183	**<0.001**
Number of children	−0.07	0.21
Self-efficacy for bicycling skills (range: 1–4)	0.14	**0.02**
AC behavioral beliefs score (range: 13–91)	0.13	**0.02**
Perceived behavioral control for AC (range: 7–42)	−0.441	**<0.001**
Interpersonal level		
Spouse AC (times/week)	0.29	**<0.001**
Spouse normative beliefs for AC (range: 4–20)	0.26	**<0.001**
Perceived coworker AC (range: 1–5)	0.07	0.13
Coworker normative beliefs for AC (range: 4–20)	0.23	**<0.001**
Institutional level		
Number of employer supports for AC (range: 0–7)	0.14	**<0.001**
Perceived employer support for AC (range: 1–5)	0.15	**<0.001**
Community level		
Perceived community support for AC (range: 5–25)	0.06	0.27
Perceived pedestrian friendliness for AC (range: 1–5)	0.1	**0.06**
Perceive bicycle friendliness for AC (range: 1–5)	0.06	0.22
Environment level (range 1–5)		
Lack of on-street bike lanes	−0.15	**0.01**
Lack of off-street walking/biking paths	−0.2	**<0.001**
Lack of sidewalks	−0.22	**<0.001**
Speed and volume of traffic along route	−0.14	**0.01**
Perceived crime along route	−0.05	0.37
Difficult terrain	−0.18	**<0.001**
Bad weather	−0.13	**0.03**

*Note*. AC: active commuting; bold face indicates significance.

**Table 3:** Hierarchical regression explaining variance in active commuting (AC).

Variable	*B*	SEB	*β*	*R* ^2^	Δ*R* ^2^
Step1: Individual influences				0.42	
Perceived health status	−0.05	0.26	−0.17		
Body mass index	0.03	0.04	0.05		
Perceived behavioral control for AC	−0.17	0.03	−0.48***		
Self-efficacy for skills	−0.07	0.2	−0.03		
Number of cars in the household	−0.3	0.27	−0.09		
Step2: Interpersonal influences				0.53	0.11
Coworker normative beliefs for AC	0.07	0.07	0.08		
Spouse AC	0.4	0.11	0.27**		
Spouse normative beliefs for AC	0.06	0.06	0.1		
Step3: Institutional influences				0.53	0.001
Perceived employer support for AC	−0.03	0.19	−0.01		
Number of employer supports for AC	0.04	0.16	0.02		
Step4: Community influences				0.56	0.03
Perceived walk time to work	−1.3	0.52	−0.1*		
Step5: Environmental influences				0.61	0.05
Lack of on-street bike lanes	0.15	0.29	0.09		
Lack of off-street walking/biking paths	0.04	0.35	0.03		
Lack of sidewalks	−0.59	0.27	−0.37*		
Speed and volume of traffic along route	0.5	0.2	0.3*		
Difficult terrain	0.15	0.2	0.08		
Bad weather	−0.23	0.19	−0.12		

*Note*. *P < 0.05, **P < 0.01, and ***P < 0.001.
